# Supplementary material for: DNA methylation signatures in peripheral blood strongly predict all-cause mortality
Source: Nat Commun. 2017 Mar 17;8:14617. doi: 10.1038/ncomms14617 (PMC5357865; doi:10.1038/ncomms14617)
Supplement: Supplementary Information — Supplementary Figures, Supplementary Tables, and Supplementary References [file ncomms14617-s1.pdf]

Supplementary Figure 1. Study design and analysis flowchart

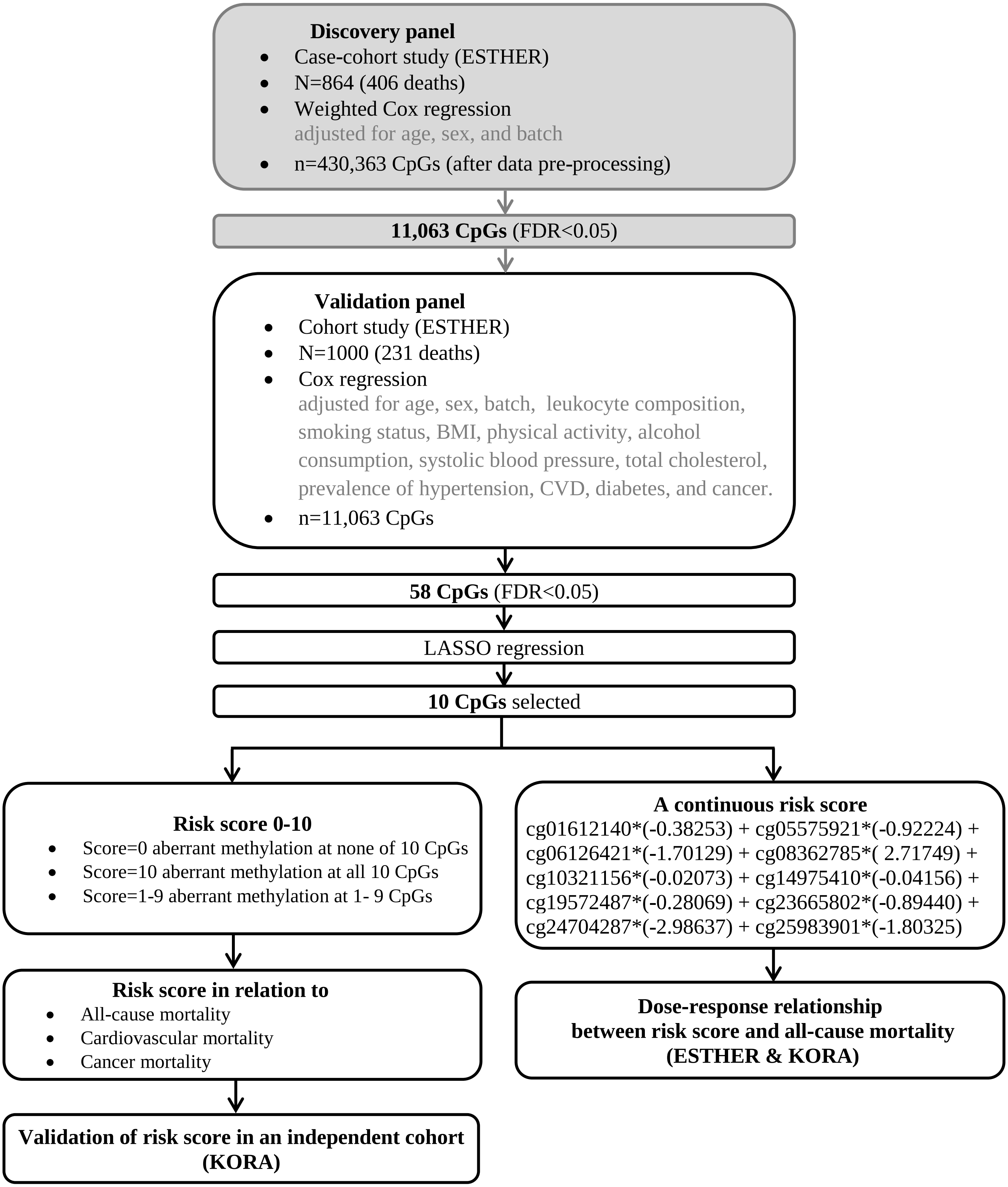

Supplementary Figure 2. Manhattan plots for the discovery (panel a; N=864) and validation (panel b; N=1000) analyses. The vertical axis indicates (-log10 transformed) observed P-values (Wald chi-square test), and the horizontal line marks the threshold of significance.

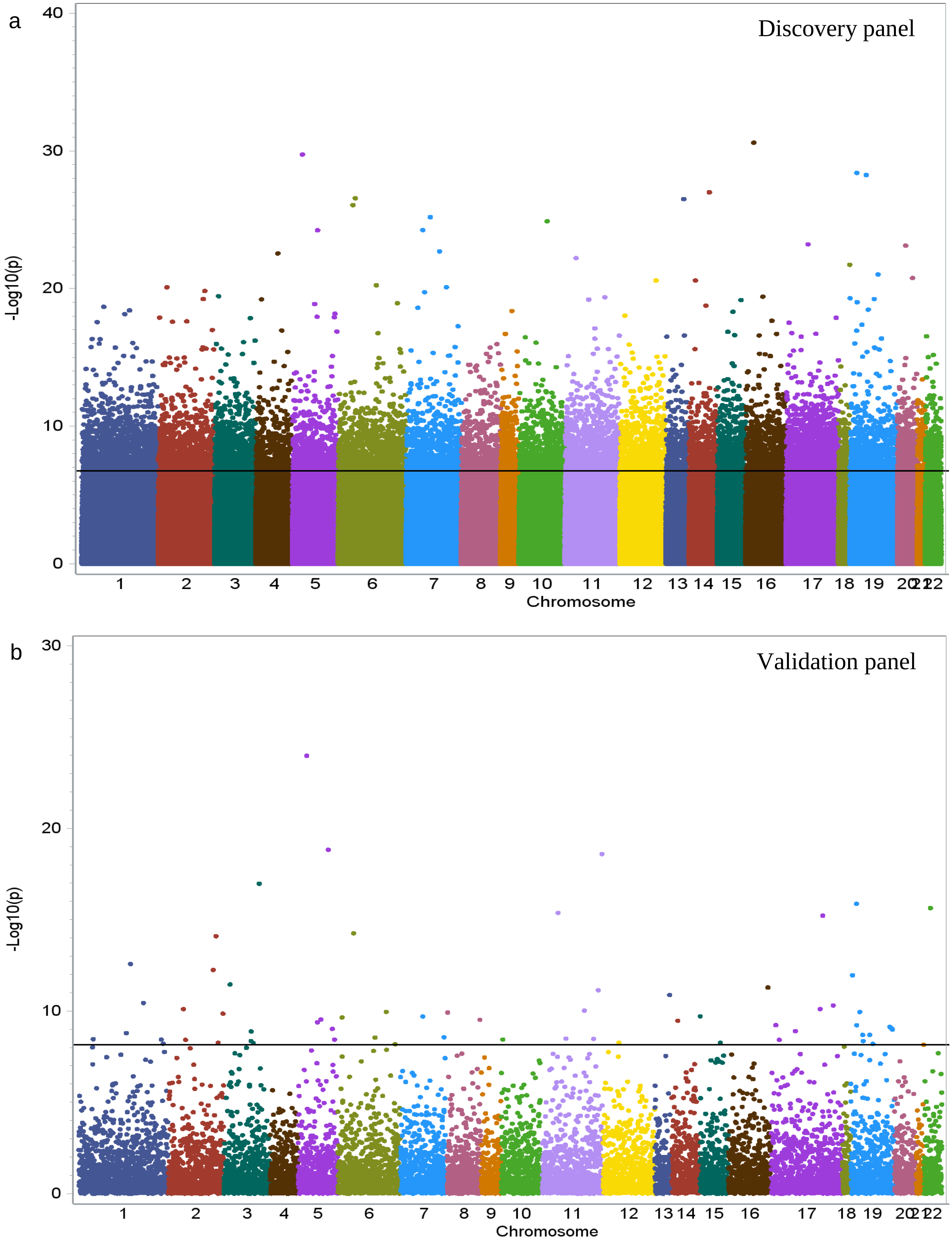

Supplementary Figure 3. CpGs associated with lifestyle factors and prevalent diseases at baseline (N=1000).

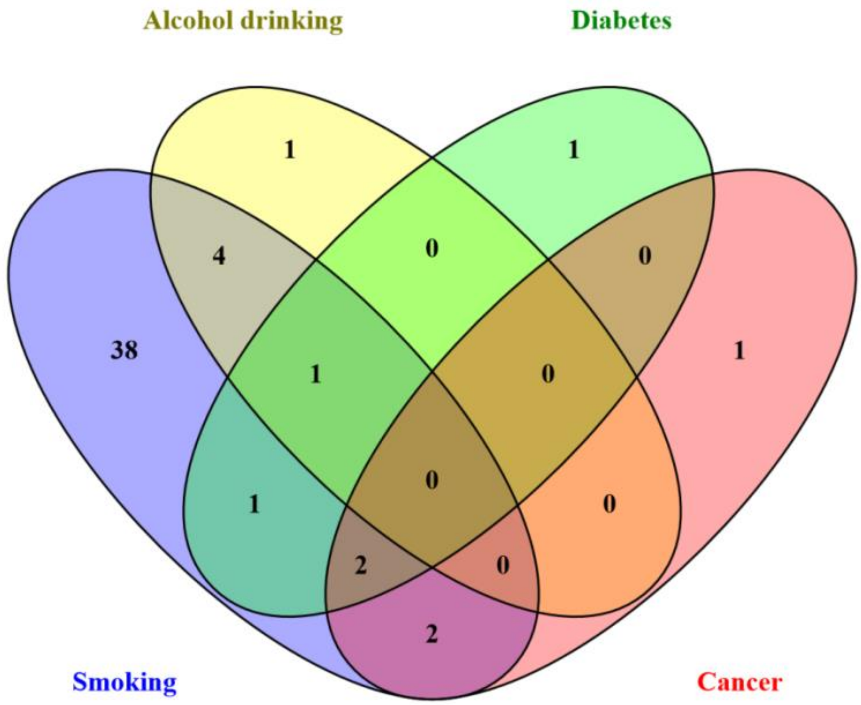

Supplementary Figure 4. Distribution of deceased across the 10 CpGs used to define the risk score in ESTHER [panel a; N=1000 (231 deaths)] and KORA study [panel b; N=1727 (61 deaths)].

a

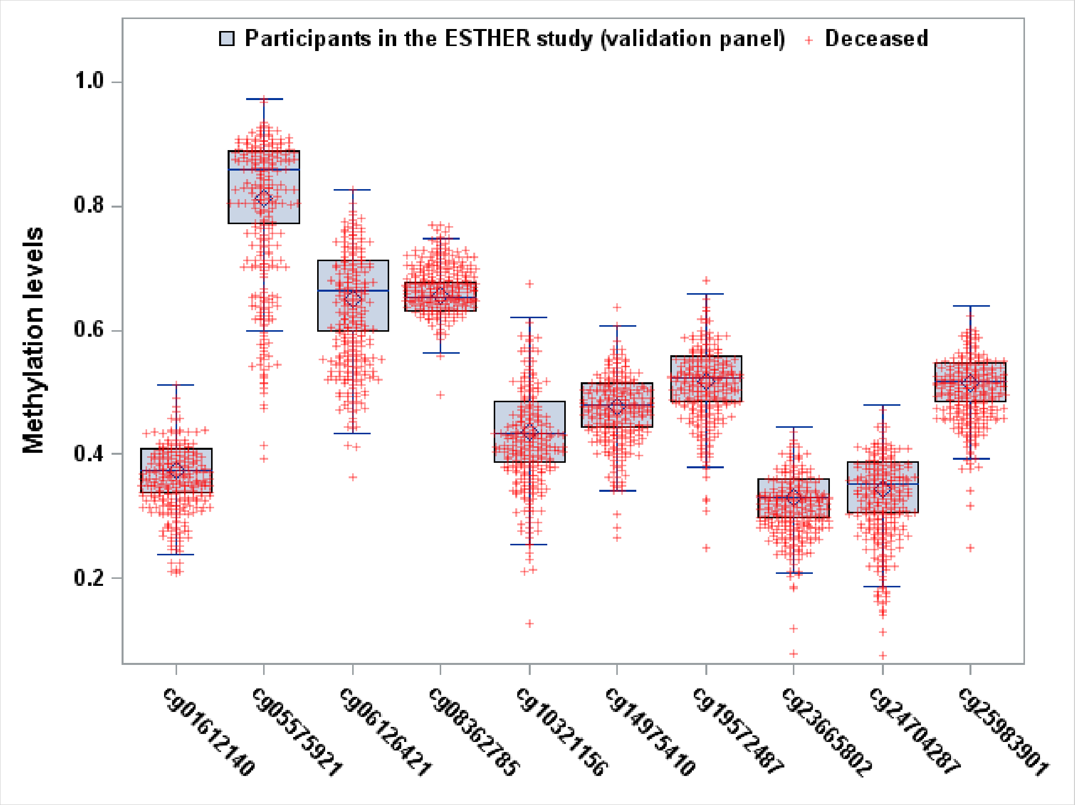

b

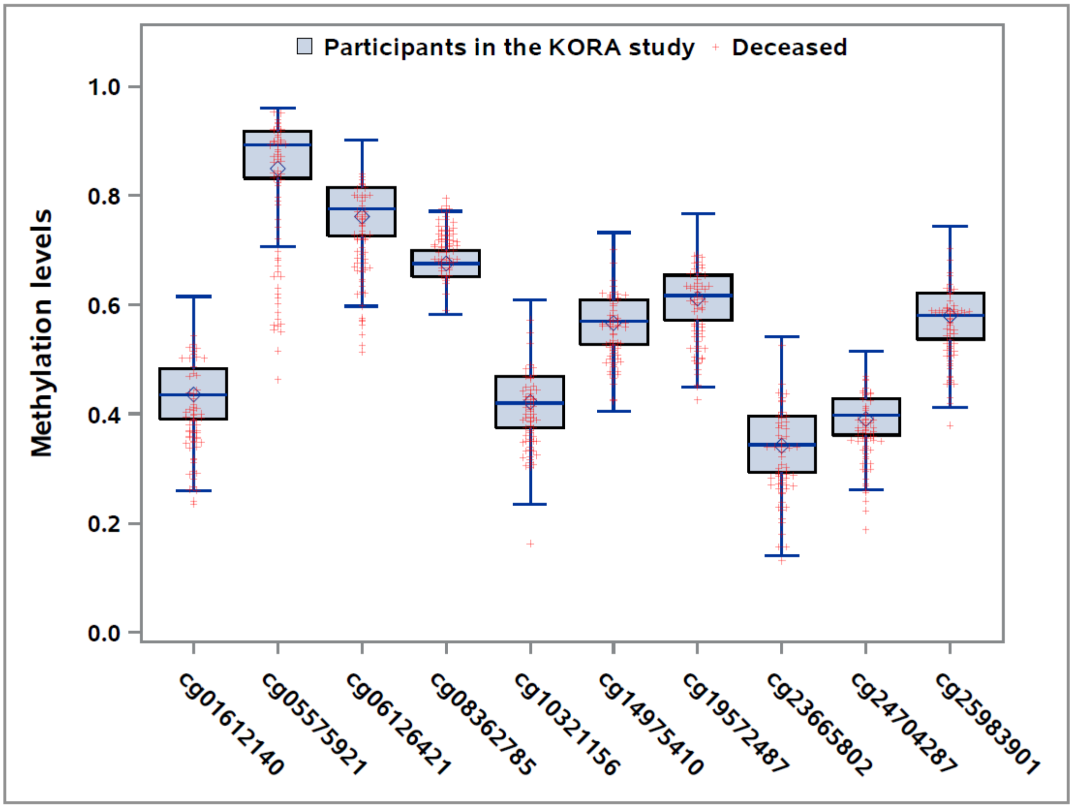

Supplementary Figure 5. Kaplan-Meier estimates of survival by risk score in the KORA study (N=1727). a. Survival curves with respect to death from any causes; b. Survival curves with respect to death from cancer; c. Survival curves with respect to death from cardiovascular disease (CVD). Plog-rank was derived from log-rank test.

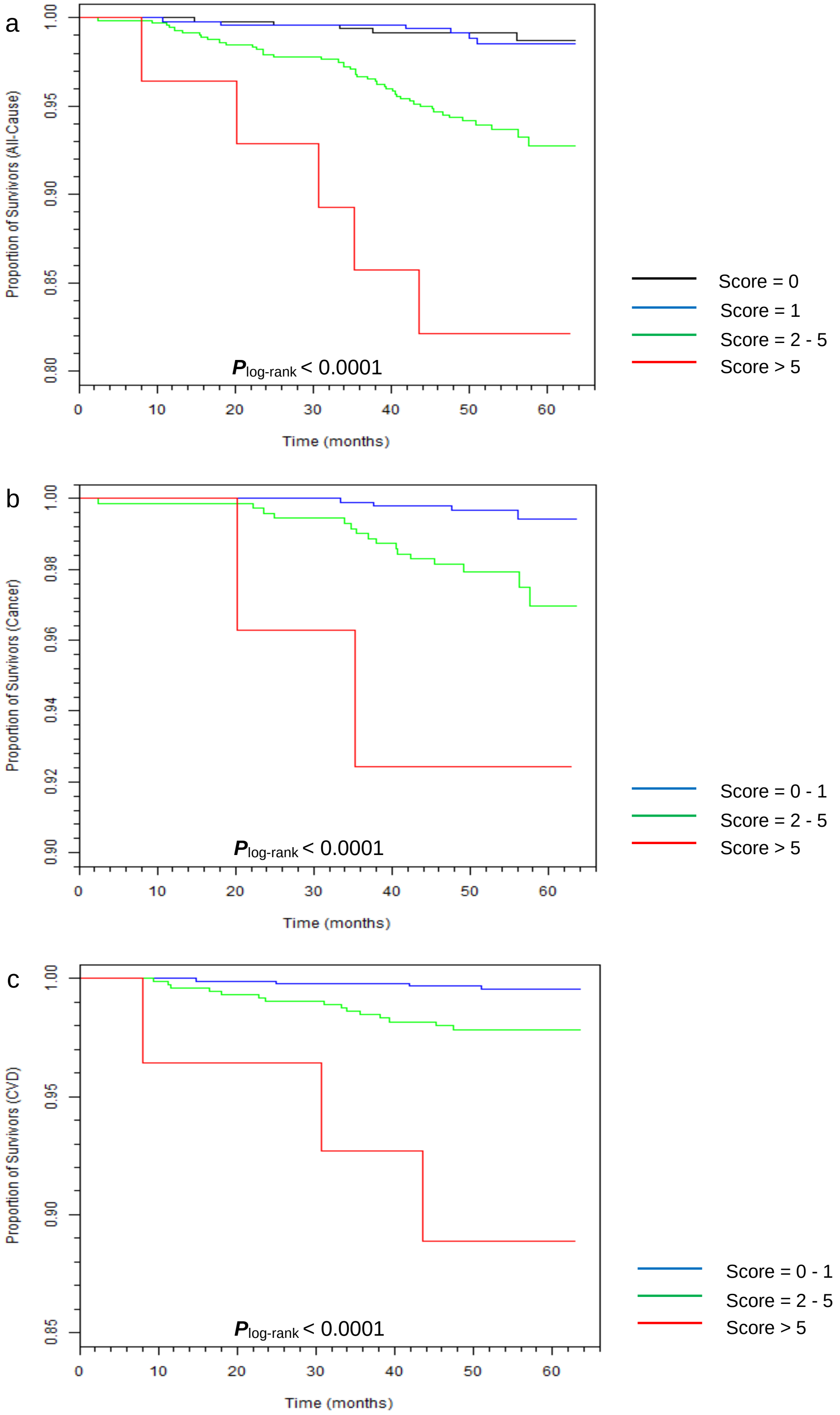

Supplementary Table 1. Characteristics of the KORA study population at baseline

Characteristic	N (%) / Mean (Standard deviation)
Male (%)	845 (48.9)
Age (years)	61.0 (8.9)
Never/ former / current smokers (%)[a]	721 (41.8) / 754 (43.7) / 250 (14.5)
Body mass index (kg/m^2)	28.1 (4.8)
Physically active (%)[a]	985 (57.1)
Hypertension (%)[b]	789 (45.8)
Diabetes (%)[c]	161 (9.3)
Coronary heart disease (%)[c]	105 (6.0)
Cancer (%)[c]	154 (8.9)
Total cholesterol (mg/dl)	221.9 (39.2)
Systolic blood pressure	124.8 (18.7)

[a]Data missing for 2 subjects; [b]Data missing for 4 subjects; [c]Data missing for 1 subject.

Supplementary Table 2. Gene functions and related diseases of identified loci

CpG site	Gene name	Chr	Gene region	Gene function and related diseases
cg03725309	*SARS*	1	Body	SARS (Seryl-TRNA Synthetase) plays a key role in vascular development,[1] and has been identified as a risk gene for coronary artery disease by genome-wide association studies.[2] Hypermethylation at cg03725309 has been observed in type 2 diabetes (T2D) patients.
cg25763716	*VCAM1*	1	TSS1500	VCAM1 (vascular cell adhesion molecule 1) plays a role in the adhesion and migration of leukocytes from blood to arterial intima and is correlate with the development of atherosclerosis.[3] VCAM-1 is over-expressed in myocardial infarction (MI) and soluble VCAM-1 (sVCAM-1) is a predictive indicator of increased risk of death in patients after MI.[4] In addition, VCAM-1 is involved in tumor invasion via mediating nerve-tumor interaction.[5]
cg25189904	*GNG12*	1	TSS1500	GNG12 [guanine nucleotide binding protein (G protein), gamma 12] is involved as a modulator or transducer in various transmembrane signaling systems. Significant differential GNG12 expression was recorded in endometrial cancer.[6]
cg15459165	*LAPTM5*	1	Body	LAPTM5 (lysosomal protein transmembrane 5) encodes a transmembrane receptor that is associated with lysosomes. Aberrant methylation at 5'-UTR of LAPTM5 and inversely correlated its gene expression have been observed in lung cancer.[7] The expression of LAPTM5 is down-regulated through DNA methylation in neuroblastoma samples and is associated with regression of neuroblastomas.[8] An earlier study demonstrated repression of LAPTM5 expression by DNA methylation in multiple myeloma.[9]
cg24397007	*FOSL2*	2	Body	FOSL2 (FOS-Like Antigen 2/ FRA2) has been implicated as a regulator of cell proliferation, differentiation, and transformation. Increased methylation of FOSL2 down-regulates the gene expression in Parkinson's disease.[10] FOSL2 gene was also found to be involved in breast cancer progression and over-expressed in endometrial cancer. [11,12]
cg16503724	*PLCL2*	3	Body	Higher methylation of PLCL2 (Phospholipase C-Like 2) and relevant down-regulation of gene expression were observed in renal cell carcinoma.[13] PLCL2 was additionally identified as a susceptibility gene for MI and systemic sclerosis.[14,15]
cg19859270	*GPR15*	3	1stExon	GPR15 (G Protein-Coupled Receptor 15) acts as a chemokine receptor for HIV.[16]
cg02657160	*CPOX*	3	Body	CPOX (Coproporphyrinogen Oxidase) encodes an enzyme that catalyzes the stepwise oxidative decarboxylation of coproporphyrinogen III to protoporphyrinogen IX, a precursor of heme. Disease associated with CPOX is hereditary coproporphyria (HCP).[17]
cg14855367	*UTS2D*	3	1stExon	UTS2D (Urotensin-2 Domain-Containing) is a protein coding gene. Urotensin II can induce endothelial independent vasoconstriction and endothelial dependent vasodilatation in vasculature. Urotensin II is regarded as a link between hypertension and coronary artery disease.[18]
cg05575921<br>cg14817490<br>cg21161138	*AHRR*<br>*AHRR*<br>*AHRR*	5<br>5<br>5	Body<br>Body<br>Body	AHRR (Aryl-Hydrocarbon Receptor Repressor), known as a tumor repressor, mediates dioxin toxicity.[19,20] AHRR methylation in blood was strongly associated with smoking-related diseases, including lung cancer,[21] atherosclerosis,[22] and death from CVD and cancer.[23]
cg20732076	*TRERF1*	6	5'UTR	TRERF1 (transcriptional regulating factor 1) encodes a zinc-finger transcriptional regulating protein which interacts with CBP/p300 to regulate the human gene CYP11A1. Diseases associated with TRERF1 include estrogen resistance and tumor aggressiveness in breast cancer patients.[24,25]

Supplementary Table 2. Continued.

CpG site	Gene name	Chr	Gene region	Gene function and related diseases
cg25285720	*HLA-DMA*	6	Body	HLA-DMA (major histocompatibility complex, class II, DM alpha) belongs to the HLA class II alpha chain paralogues. This class II molecule is a heterodimer consisting of an alpha (DMA) and a beta chain (DMB), both anchored in the membrane. It is located in intracellular vesicles. Tumor hypomethylation of HLA-DMA associates with longer time to recurrence of high-grade serous epithelial ovarian cancer.[26]
cg06126421		6		Cg06126421 is located at an intergenic region of 6p21.33. Smoking-induced hypomethylation of this locus was found to be associated with lung cancer incidence,[21] and death from cancer and CVD.[23]
cg12510708	*NFE2L3*	7	Body	NFE2L3 (nuclear factor, erythroid 2-Like 3) encodes a protein that heterodimerizes with small musculoaponeurotic fibrosarcoma factors to bind antioxidant response elements in target genes. NFE2L3 was disclosed to display hypermethylation for estrogen receptor/progesterone receptor (ER/PR) positive breast cancer and hypomethylation for ER/PR negative breast cancer.[27] An association of NFE2L3 methylation with T2D was recently reported.[28]
cg26286961	*CSGALNACT1*	8	TSS200	CSGALNACT1 (chondroitin sulfate N-acetylgalactosaminyltransferase 1) is a critical glycosyltransferase of chondroitin sulfate biosynthesis. CSGALNACT1 is significantly downregulated in the follicular variant of papillary thyroid carcinoma (FV-PTC) and high expression of CSGALNACT1 was associated with favorable prognosis of multiple myeloma.[29,30] This gene was suggested to play roles in Kashin-Beck disease and primary osteoarthritis.[31]
cg00285394	*SQLE*	8	Body	SQLE (squalene epoxidase) catalyzes the first oxygenation step in sterol biosynthesis. Expression of SQLE was found to be associated with ER+ breast cancer and inversely related to distant metastasis-free survival in stage I/II breast cancer patients. [32,33] Overexpression of SQLE promotes cell proliferation and migration of the hepatocellular carcinoma cells.[34] SQLE plays roles in lung cancer and prostate cancer.[35,36] SQLE was also identified to belong to a network of coexpressed cholesterol metabolism genes and involved in obesity-related T2D and CVD.[37]
cg01140244	*INPP5A*	10	Body	INPP5A (inositol polyphosphate-5-phosphatase A) was suggested to play a role of tumor suppressor. Loss of INPP5A was found to be associated with brain tumors.[38,39] INPP5A was also identified to be involved in development and progression of cutaneous squamous cell carcinoma.[40]
cg23190089	*SLC22A18AS*	11	Body	SLC22A18AS (solute carrier family 22 member 18 antisense) is the antisense partner of SLC22A18. Gain of imprinting of both the genes has been observed in breast cancer and diabetes.[41,42] The expression of SLC22A18AS was found to be methylation dependent.[43]
cg07123182<br>cg26963277	*KCNQ1OT1*<br>*KCNQ1OT1*	11<br>11	TSS1500<br>TSS1500	KCNQ1OT1 (KCNQ1 opposite strand/antisense transcript 1) is the antisense to the KCNQ1 gene and is a unspliced long non-coding RNA. It interacts with chromatin and regulates transcription of multiple target genes through epigenetic modifications. The KCNQ1OT1 transcript plays an important role in colorectal/breast carcinogenesis and mediates diabetes susceptibility.[42,44-46] KCNQ1OT1 was also suggested to improve the prediction of left ventricular dysfunction.[47]
cg18550212	*ATL3*	11	Body	ATL3 (Atlastin GTPase 3) encodes a protein which is required for the proper formation of the network of interconnected tubules of the endoplasmic reticulum. It was identified to be associated with neuropathy.[48]

Supplementary Table 2. Continued.

CpG site	Gene name	Chr	Gene region	Gene function and related diseases
cg25193885	*SHANK2*	11	Body	SHANK2 (SH3 and multiple ankyrin repeat domains 2) encodes a protein that is a member of the Shank family of synaptic proteins that has been suggested to play roles in neuropsychiatric disorders.[49] A recent epigenome-wide association study (EWAS) identified promoter region of SHANK2 to be differentially methylated in prostate cancer tissues compare to normal controls.[50] Another EWAS disclosed that SHANK2 methylation was involved in intellectual disability.[51]
cg07986378	*ETV6*	12	Body	ETV6 (ETS variant 6) encodes an ETS family transcription factor. This gene is known to be involved in a large number of chromosomal rearrangements associated with leukemia and congenital fibrosarcoma.[52] Genetic studies identified a central role for ETV6 in hematopoiesis and malignant transformation.[53]
cg23665802	*MIR19A*	13	TSS1500	MIR19A (MicroRNA 19a) is an RNA gene, and is affiliated with the miRNA class. MicroRNAs are involved in cancer pathways, regulating gene expression by affecting stability and translation of target mRNAs. MIR19A has been suggested functioning as an oncogenic microRNA and is up-regulated in a variety of malignant conditions, including lung cancer, colorectal, gastric, breast, bladder, cervical, and esophageal cancer, as well as hepatocellular carcinoma, and leukemia.[54-62] Circulating MIR19A levels was found to be higher in acute MI and suggested to be a potential candidate for diagnosis of acute MI.[63]
cg04987734	*CDC42BPB*	14	Body	CDC42BPB (CDC42 binding protein kinase beta) encodes a member of the serine/threonine protein kinase family. CDC42BPB is a key regulator of cancer cell migration and invasion and has been identified as a novel potential therapeutic/drug target for cancer, such as colorectal and breast cancer.[64-66]
cg00310412	*SEMA7A*	15	Body	SEMA7A (semaphorin 7A) encodes a protein that is found on activated lymphocytes and erythrocytes and might be involved in immunomodulatory and neuronal processes. It has been suggested that gene products of SEMA7A play a role as pooled cerebrospinal fluid biomarkers that were associated with the conversion to clinically definite multiple sclerosis.[67] SEMA7A may also be involved in chronic asthma through regulating eosinophil profibrotic functions in the airway, and contribute to liver fibrogenesis.[68,69]
cg26709988	*CRISPLD2*	16	5'UTR	CRISPLD2 (cysteine rich secretory protein LCCL domain containing 2) was suggested as a target of the progesterone receptor and may play an important role in pathogenesis of endometriosis.[70]
cg23842572	*MPRIP*	17	Body	MPRIP (myosin phosphatase Rho interacting protein) plays a critical role for human cancer cell invasion.[71]
cg19572487	*RARA*	17	5'UTR	RARA (retinoic acid receptor alpha) has been implicated in regulation of development, differentiation, apoptosis, granulopoeisis, and transcription of clock genes. Translocations between this locus and several other loci have been found to be associated with hematologic neoplasms, mammary tumorigenesis, as well as hepatocellular and thyroid carcinomas.[72-75]
cg01572694	*MIR10A*	17	TSS1500	MIR10A (MicroRNA 10a) is an RNA gene, and is affiliated with the miRNA class. MIR10A has been disclosed to be involved in development and progression of lung, breast, gastric, pancreatic, colon, brain, bladder, cervical, thyroid, esophageal cancer, as well as HCC, melanoma, leukemia.[76-84] A recent study combining next-generation sequencing omics data with gene regulatory networks, microRNAs regulatory networks, protein-protein interaction networks, and epigenetic regulatory networks of methylation suggested that MIR10A contributes to the HIV-1 integration and replication stage.[85]

Supplementary Table 2. Continued.

CpG site	Gene name	Chr	Gene region	Gene function and related diseases
cg08546016	*TMEM104*	17	Body	TMEM104 (transmembrane protein 104) is a protein coding gene.
cg18181703	*SOCS3*	17	Body	SOCS3 (suppressor of cytokine signaling 3) encodes a member of the suppressor of cytokine signaling family. The expression of this gene is induced by various cytokines, including IL6, IL10, and interferon (IFN)-gamma. Aberrant methylation of SOCS3 plays roles in tumorigenesis, including lung, pancreatic, colorectal, head and neck, prostate, cervical, and endometrial cancer, as well as hepatocellular carcinoma, melanoma, glioblastoma, and leukemia.[86-96] Three recent EWAS studies identified that SOCS3 methylation was associated with incidence of T2D. [28,97,98]
cg03636183	*F2RL3*	19	Body	F2RL3 encodes for the thrombin protease-activated receptor-4 (PAR-4) that plays roles in blood coagulation and inflammatory reactions.[99] PAR4 is over-expressed in various malignant conditions, such as colorectal cancer, and prostate cancer. F2RL3 demethylation is strongly predictive for lung cancer incidence and mortality, as well as CVD and cancer mortality.[21,23,100]
cg11341610	*CALR*	19	Body	CALR (calreticulin) is a multifunctional protein that acts as a major Ca^{2+}-binding protein in the lumen of the endoplasmic reticulum. There is accumulating evidence that CALR plays roles in the development and progression of various cancers, e.g., lung, gastric, pancreatic, prostate, ovarian, esophageal cancers, neuroblastoma, and leukemia.[101-104]
cg26470501	*BCL3*	19	Body	BCL3 (B-cell CLL/lymphoma 3) is a proto-oncogene candidate and protects cells from apoptosis. BCL3 is an established oncogene in hematologic malignancies, such as B-cell chronic lymphocytic leukemia.[105] Recent studies have shown that it also participates in progression of various solid tumors, including cancers in lung, breast, colorectal, and prostate.[106-109] BCL3 was additionally suggested to be involved in pathogenesis of CVD (such as atherosclerosis and MI),[110,111] and rheumatoid arthritis.[112]
cg05492306	*ERCC1*	19	TSS1500	ERCC1(excision repair cross-complementation group 1) encodes a protein that is required for the repair of DNA lesions such as those induced by UV light or formed by electrophilic compounds including cisplatin. This gene was identified to be involved in malignant conditions, including lung, breast, and gastric cancer, as well as head and neck squamous cell carcinoma. [113-117]
cg25607249<br>cg01406381<br>cg07626482	*SLC1A5*<br>*SLC1A5*<br>*SLC1A5*	19<br>19<br>19	1stExon<br>TSS200<br>TSS1500	SLC1A5 (solute carrier family 1 member 5/ ASCT2) is a high-affinity transporter of glutamine in various cancer. It has been demonstrated that SLC1A5 expression is upregulated in lung, colorectal, breast, prostate, pancreatic cancer, neuroblastoma, melanoma, and renal cell carcinoma.[118-125] Differential DNA methylation concurrent differentially mRNA expression of SLC1A5 has been observed in pancreatic islets for T2D patients compared to non-diabetic controls.[126]
cg03707168	*PPP1R15A*	19	Body	PPP1R15A (protein phosphatase 1 regulatory subunit 15A/ GADD34) is a member of a group of genes whose transcript levels are increased following stressful growth arrest conditions and treatment with DNA-damaging agents. PPP1R15A was found to play roles in neurological diseases, myocardial ischaemia.[127,128] It has been observed that PPP1R15A-deficient mice develop obesity, nonalcoholic fatty liver disease, hepatic carcinoma, insulin resistance, and colon cancer.[129,130]

Supplementary Table 2. Continued.

CpG site	Gene name	Chr	Gene region	Gene function and related diseases
cg25491402	*PDE9A*	21	Body	PDE9A (phosphodiesterase 9A) encodes a protein that plays a role in signal transduction by regulating the intracellular concentration of these cyclic nucleotides. PDE9A is involved in stress-induced heart disease.[131] Overexpression of PDE9A was also identified in breast cancer.[132] DNA hypermethylation of PDE9A resulted in reduced mRNA expression in lung tumor tissue.[133]
cg08362785	*MKL1*	22	Body	MKL1[megakaryoblastic leukemia (translocation) 1/ MRTF-A] encodes a protein that is predominantly nuclear and may help to transduce signals from the cytoskeleton to the nucleus. MKL1 was shown to enhance lung and breast cancer cell migration and invasion by epigenetic modifications.[134,135] MKL1 additionally plays roles in lung and liver fibrosis.[136,137]

Supplementary Table 3. List of CpGs associated with lifestyle factors and prevalent diseases at baseline

		FDR[a]					
CpG site	Gene name	sex (n=23 CpGs)	age (n=23 CpGs)	smoking status (n=48 CpGs)	alcohol consumption (n=6 CpGs)	diabetes (n=5 CpGs)	Cancer (n=5 CpGs)
cg03725309	*SARS*		4.25E-08	0.0138	2.24E-05		
cg25763716	*VCAM1*		0.0022				
cg13854219				0.0003			
cg25189904	*GNG12*	2.19E-08		4.47E-25			
cg15459165	*LAPTM5*			0.0007			
cg19266329		0.0133		5.38E-05	0.0008	0.0014	
cg24397007	*FOSL2*				0.0002		
cg23079012		9.99E-05		9.59E-24			
cg27241845		2.67E-27		6.50E-23			
cg06905155							0.0080
cg16503724	*PLCL2*	0.0232		3.95E-12			
cg19859270	*GPR15*	0.0029		1.35E-51			
cg02657160	*CPOX*			1.75E-16			
cg14975410			0.0021	0.0033			
cg05575921	*AHRR*	2.69E-10		2.18E-135			
cg14817490	*AHRR*		0.0370	6.35E-52			
cg21161138	*AHRR*	0.0063		3.01E-69			
cg12513616		2.19E-08		9.42E-18			
cg20732076	*TRERF1*		0.0283	3.52E-09	0.0230		
cg06126421		5.92E-08	0.0198	1.79E-61			
cg15342087		0.0028		3.84E-40			
cg01612140		6.95E-08		0.0103			
cg25983901			0.0194	0.0015	0.0002		
cg12510708	*NFE2L3*		0.0021	0.0093			
cg00285394	*SQLE*	0.0017		0.0021			
cg01140244	*INPP5A*		0.0022				
cg23190089	*SLC22A18AS*	0.0014	1.74E-05	2.14E-09		0.0199	
cg07123182	*KCNQ1OT1*		0.0198	2.58E-17			

Supplementary Table 3. Continued.

		FDR[a]					
CpG site	Gene name	sex (n=23 CpGs)	age (n=23 CpGs)	smoking status (n=48 CpGs)	alcohol consumption (n=6 CpGs)	diabetes (n=5 CpGs)	Cancer (n=5 CpGs)
cg26963277	*KCNQ1OT1*	0.0104		1.51E-21			
cg18550212	*ATL3*		0.0021	0.0277			
cg10321156		6.93E-06		0.0173			
cg25193885	*SHANK2*	0.0422		0.0372			
cg07986378	*ETV6*	0.0488		7.51E-20			
cg23665802	*MIR19A*		0.0004	3.74E-05			
cg04987734	*CDC42BPB*	0.0169		0.0050			
cg19459791				2.32E-05			
cg00310412	*SEMA7A*	0.0002		1.62E-20			
cg23842572	*MPRIP*		0.0370	5.85E-06			
cg19572487	*RARA*	4.68E-10	0.0018	7.66E-36			
cg01572694	*MIR10A*			0.0479			
cg08546016	*TMEM104*			0.0338			
cg18181703	*SOCS3*			0.0125		0.0373	0.0303
cg03636183	*F2RL3*	0.00012		1.01E-95			0.0145
cg24704287			0.0030	3.85E-07			0.0117
cg11341610	*CALR*	0.0187	0.0026	0.0021			
cg14085840		0.0492		0.0003			
cg26470501	*BCL3*		0.0038	6.77E-06		0.0199	0.0117
cg05492306	*ERCC1*		8.84E-05	2.27E-06			
cg25607249	*SLC1A5*		0.0407	9.34E-05			
cg01406381	*SLC1A5*		0.0070	5.85E-06			
cg07626482	*SLC1A5*			2.32E-05	5.53E-07		
cg03707168	*PPP1R15A*		0.0030	2.70E-31			
cg08362785	*MKL1*	1.02E-05	2.05E-07			0.0445	

[a] FDR, False discovery rate; FDR<0.05 in mixed regression analysis with each validated CpG as dependent variable and age (years), sex, smoking status (never/former/current smokers), alcohol consumption, body mass index ((<25 kg/m2 / 25.0-<30.0 kg/m2 / ≥30.0 kg/m2), physical activity (inactive/low/medium or high), total cholesterol, prevalence of hypertension, cardiovascular disease, diabetes, and cancer as independent variables, controlling for batch effects and leukocyte composition.

Supplementary Table 4. Cutoff points for defining aberrant methylation for the 10 CpG

CpG sites	cutoff points
cg01612140	≤ 0.33962
cg05575921	≤ 0.77332
cg06126421	≤ 0.59937
cg08362785	≥ 0.67841
cg10321156	≤ 0.38737
cg14975410	≤ 0.44420
cg19572487	≤ 0.48629
cg23665802	≤ 0.29927
cg24704287	≤ 0.30730
cg25983901	≤ 0.48522

Supplementary Table 5. Sex-specific associations of risk score with all-cause mortality in the ESTHER study and KORA study

Group	Mortality score[a]	N_{total}	Cases	PY	IR[b]	HR (95% CI)		
						Model 1[c]	Model 2[d]	Model 3[e]
ESTHER-Men	0	61	4	808.09	0.50	Ref.	Ref.	Ref.
	1	97	23	1217.62	1.89			
	2-5	236	62	2888.82	2.15	1.63 (1.04 – 2.55)	1.51 (0.96 – 2.37)	1.50 (0.93 – 2.43)
	>5	105	54	1068.33	5.05	3.94 (2.48 – 6.26)	3.32 (2.08 – 5.29)	3.04 (1.76 – 5.25)
ESTHER-Women	0	138	10	1882.60	0.53	Ref.	Ref.	Ref.
	1	145	18	1926.88	0.93			
	2-5	189	43	2412.04	1.78	2.51 (1.56 – 4.05)	2.48 (1.54 – 3.99)	2.67 (1.59 – 4.48)
	>5	26	16	280.65	5.70	9.17 (4.95 – 16.98)	8.36 (4.50 – 15.51)	8.31 (3.76 – 18.35)
KORA-Men	0	177	1	795.15	0.13	Ref.	Ref.	Ref.
	1	220	3	958.78	0.31			
	2-5	428	36	1810.53	1.99	8.86 (3.15 - 24.9)	7.16 (2.53 - 20.3)	6.17 (2.13 - 17.84)
	>5	20	4	80.41	4.97	22.28 (5.57 - 89.1)	16.9 (4.19 - 68.19)	10.46 (2.26 - 48.41)
KORA-Women	0	310	4	1367.86	0.29	Ref.	Ref.	Ref.
	1	270	3	1189.13	0.25			
	2-5	294	9	1259.57	0.71	2.61 (0.97 - 7.01)	2.1 (0.78 - 5.66)	1.86 (0.61 - 5.63)
	>5	8	1	33.91	2.95	10.85 (1.33 - 88.21)	17.73 (2.08 - 151.49)	14.34 (1.02 - 201.11)

Abbreviations: HR, hazard ratio; CI, confidence interval; CVD, cardiovascular disease; IR, incidence rate; PY, person-years; Ref., reference category.

[a] Score was based on methylation of 10 CpGs (cg01612140, cg05575921, cg06126421, cg08362785, cg10321156, cg14975410, cg19572487, cg23665802, cg24704287, cg25983901) using their respective first quartile values (cg08362785: using its highest quartile) among the ESTHER participants as the cutoff points to define aberrant methylation; Score 0-10 refer to simultaneously aberrant methylation at 0 to 10 CpGs; [b]Incidence rate per 100 person-years; [c]Model 1: without adjustment; [d]Model 2: adjusted for chronological age. [f]Model 3: like model 2, additionally adjusted for smoking status, BMI, physical activity, alcohol consumption, systolic blood pressure, total cholesterol, hypertension, and prevalent cardiovascular disease, diabetes, and cancer at baseline.

Supplementary Table 6. Associations of the risk score and epigenetic clock (calculated according to Horvath's algorithm) with all-cause and cause-specific mortality in the ESTHER study

Outcome	Mortality score[a] / epigenetic clock[b]	HR (95% CI)		
		Model 1[c]	Model 2[d]	Model 3[e]
All-cause mortality	0	Ref.	Ref.	Ref.
	1	2.04 (1.11 – 3.75)	2.04 (1.11 – 3.75)	2.18 (1.11 – 4.28)
	2-5	3.18 (1.81 – 5.59)	3.15 (1.78 – 5.55)	3.45 (1.82 – 6.54)
	>5	7.64 (4.21 – 13.85)	7.55 (4.14 – 13.76)	7.47 (3.72 – 15.00)
	Horvath Δage (per 5 years)	1.14 (1.00 – 1.30)	1.02 (0.90 – 1.16)	0.98 (0.85 – 1.13)
Cancer mortality	0<br>1	Ref.	Ref.	Ref.
	2-5	1.24 (0.72 – 2.11)	1.19 (0.69 – 2.05)	1.19 (0.67 – 2.12)
	>5	3.12 (1.69 – 5.78)	2.96 (1.58 – 5.54)	2.49 (1.21 – 5.14)
	Horvath Δage (per 5 years)	1.18 (0.95 – 1.47)	1.10 (0.88 – 1.38)	1.04 (0.82 – 1.32)
CVD mortality	0<br>1	Ref.	Ref.	Ref.
	2-5	3.41 (1.82 – 6.40)	3.40 (1.80 – 6.42)	3.99 (1.94 – 8.20)
	>5	7.19 (3.54 – 14.62)	7.17 (3.48 – 14.74)	9.10 (3.81 – 21.71)
	Horvath Δage (per 5 years)	1.15 (0.93 – 1.43)	1.00 (0.81 – 1.25)	1.00 (0.78 – 1.28)

Abbreviations: HR, hazard ratio; CI, confidence interval; CVD, cardiovascular disease; Ref., reference category.

[a]Score was based on methylation of 10 CpGs (cg01612140, cg05575921, cg06126421, cg08362785, cg10321156, cg14975410, cg19572487, cg23665802, cg24704287, cg25983901) using their respective first quartile values (cg08362785: using its highest quartile) among the ESTHER participants as the cutoff points to define aberrant methylation; Score 0-10 refer to simultaneously aberrant methylation at 0 to 10 CpGs; [b]The epigenetic clock estimated by the difference between DNA methylation calculated according to Horvath's algorithm[138] and chronological age; [c]Model 1: adjusted for age and sex; [d]Model 2: like model 1, additionally adjusted for the epigenetic clock/risk score; [e]Model 3: like model 2, additionally adjusted for smoking status, BMI, physical activity, alcohol consumption, systolic blood pressure, total cholesterol, hypertension, and prevalent cardiovascular disease, diabetes, and cancer at baseline.

Supplementary References

1 Herzog, W., Muller, K., Huisken, J. & Stainier, D. Y. Genetic evidence for a noncanonical function of seryl-tRNA synthetase in vascular development. *Circ Res*. **104**, 1260-1266 (2009).

2 Steenaard, R. V. *et al.* Tobacco smoking is associated with methylation of genes related to coronary artery disease. *Clin Epigenetics*. **7**, 54 (2015).

3 Lisowska, A. *et al.* sVCAM-1 concentration and carotid IMT values in patients with acute myocardial infarction--Atherosclerotic markers of the presence, progress and prognosis. *Adv Med Sci*. **60**, 101-106 (2015).

4 Radecke, C. E. *et al.* Coronary artery endothelial cells and microparticles increase expression of VCAM-1 in myocardial infarction. *Thromb Haemost*. **113**, 605-616 (2015).

5 Xia, Q. *et al.* Interaction Between Gastric Carcinoma Cells and Neural Cells Promotes Perineural Invasion by a Pathway Involving VCAM1. *Dig Dis Sci*. **60**, 3283-3292 (2015).

6 Orchel, J. *et al.* Expression patterns of kinin-dependent genes in endometrial cancer. *Int J Gynecol Cancer*. **22**, 937-944 (2012).

7 Cortese, R., Hartmann, O., Berlin, K. & Eckhardt, F. Correlative gene expression and DNA methylation profiling in lung development nominate new biomarkers in lung cancer. *Int J Biochem Cell Biol*. **40**, 1494-1508 (2008).

8 Inoue, J. *et al.* Lysosomal-associated protein multispanning transmembrane 5 gene (LAPTM5) is associated with spontaneous regression of neuroblastomas. *PLoS One*. **4**, e7099 (2009).

9 Hayami, Y. *et al.* Inactivation of the E3/LAPTm5 gene by chromosomal rearrangement and DNA methylation in human multiple myeloma. *Leukemia*. **17**, 1650-1657 (2003).

10 Fernandez-Santiago, R. *et al.* Aberrant epigenome in iPSC-derived dopaminergic neurons from Parkinson's disease patients. *EMBO Mol Med*. **7**, 1529-1546 (2015).

11 Milde-Langosch, K. *et al.* Role of Fra-2 in breast cancer: influence on tumor cell invasion and motility. *Breast Cancer Res Treat*. **107**, 337-347 (2008).

12 Bamberger, A. M., Milde-Langosch, K., Rossing, E., Goemann, C. & Loning, T. Expression pattern of the AP-1 family in endometrial cancer: correlations with cell cycle regulators. *J Cancer Res Clin Oncol*. **127**, 545-550 (2001).

13 Dmitriev, A. A. *et al.* Epigenetic alterations of chromosome 3 revealed by NotI-microarrays in clear cell renal cell carcinoma. *Biomed Res Int*. **2014**, 735292 (2014).

14 Hirokawa, M. *et al.* A genome-wide association study identifies PLCL2 and AP3D1-DOT1L-SF3A2 as new susceptibility loci for myocardial infarction in Japanese. *Eur J Hum Genet*. **23**, 374-380 (2015).

15 Arismendi, M. *et al.* Identification of NF-kappaB and PLCL2 as new susceptibility genes and highlights on a potential role of IRF8 through interferon signature modulation in systemic sclerosis. *Arthritis Res Ther*. **17**, 71 (2015).

16 Blaak, H. *et al.* CCR5, GPR15, and CXCR6 are major coreceptors of human immunodeficiency virus type 2 variants isolated from individuals with and without plasma viremia. *J Virol*. **79**, 1686-1700 (2005).

17 Bissell, D. M., Wang, B. & Lai, J. in *GeneReviews(R)* (eds R. A. Pagon *et al.*) (1993).

18 Watanabe, T., Kanome, T., Miyazaki, A. & Katagiri, T. Human urotensin II as a link between hypertension and coronary artery disease. *Hypertens Res*. **29**, 375-387 (2006).

19 Zudaire, E. *et al.* The aryl hydrocarbon receptor repressor is a putative tumor suppressor gene in multiple human cancers. *J Clin Invest*. **118**, 640-650 (2008).

20 Kasai, A. *et al.* High levels of dioxin-like potential in cigarette smoke evidenced by in vitro and in vivo biosensing. *Cancer Res*. **66**, 7143-7150 (2006).

21 Fasanelli, F. *et al.* Hypomethylation of smoking-related genes is associated with future lung cancer in four prospective cohorts. *Nat Commun*. **6**, 10192 (2015).

22 Reynolds, L. M. *et al.* DNA Methylation of the Aryl Hydrocarbon Receptor Repressor Associations With Cigarette Smoking and Subclinical Atherosclerosis. *Circ Cardiovasc Genet*. **8**, 707-716 (2015).

23 Zhang, Y. *et al.* Smoking-Associated DNA Methylation Biomarkers and Their Predictive Value for All-Cause and Cardiovascular Mortality. *Environ Health Perspect*. **124**, 67-74 (2016).

24 Gizard, F. *et al.* TReP-132 is a novel progesterone receptor coactivator required for the inhibition of breast cancer cell growth and enhancement of differentiation by progesterone. *Mol Cell Biol*. **26**, 7632-7644 (2006).

25 van Agthoven, T. *et al.* Relevance of breast cancer antiestrogen resistance genes in human breast cancer progression and tamoxifen resistance. *J Clin Oncol*. **27**, 542-549 (2009).

26 Wang, C. *et al.* Tumor hypomethylation at 6p21.3 associates with longer time to recurrence of high-grade serous epithelial ovarian cancer. *Cancer Res*. **74**, 3084-3091 (2014).

27 Rauscher, G. H. *et al.* Exploring DNA methylation changes in promoter, intragenic, and intergenic regions as early and late events in breast cancer formation. *BMC Cancer*. **15**, 816 (2015).

28 Kulkarni, H. *et al.* Novel epigenetic determinants of type 2 diabetes in Mexican-American families. *Hum Mol Genet*. **24**, 5330-5344 (2015).

29 Schulten, H. J. *et al.* Comparison of microarray expression profiles between follicular variant of papillary thyroid carcinomas and follicular adenomas of the thyroid. *BMC Genomics*. **16 Suppl 1**, S7 (2015).

30 Agnelli, L. *et al.* The reconstruction of transcriptional networks reveals critical genes with implications for clinical outcome of multiple myeloma. *Clin Cancer Res*. **17**, 7402-7412 (2011).

31 Zheng, J. *et al.* Abnormal expression of chondroitin sulphate N-acetylgalactosaminyltransferase 1 and Hapln-1 in cartilage with Kashin-Beck disease and primary osteoarthritis. *Int Orthop*. **37**, 2051-2059 (2013).

32 Helms, M. W. *et al.* Squalene epoxidase, located on chromosome 8q24.1, is upregulated in 8q+ breast cancer and indicates poor clinical outcome in stage I and II disease. *Br J Cancer*. **99**, 774-780 (2008).

33 Parris, T. Z. *et al.* Frequent MYC coamplification and DNA hypomethylation of multiple genes on 8q in 8p11-p12-amplified breast carcinomas. *Oncogenesis*. **3**, e95 (2014).

34 Sui, Z., Zhou, J., Cheng, Z. & Lu, P. Squalene epoxidase (SQLE) promotes the growth and migration of the hepatocellular carcinoma cells. *Tumour Biol*. **36**, 6173-6179 (2015).

35 Zhang, H. Y., Li, H. M., Yu, Z., Yu, X. Y. & Guo, K. Expression and significance of squalene epoxidase in squamous lung cancerous tissues and pericarcinoma tissues. *Thorac Cancer*. **5**, 275-280 (2014).
36 Zhao, S. G. *et al.* The Landscape of Prognostic Outlier Genes in High-Risk Prostate Cancer. *Clin Cancer Res* (2015).
37 Ding, J. *et al.* Alterations of a Cellular Cholesterol Metabolism Network Are a Molecular Feature of Obesity-Related Type 2 Diabetes and Cardiovascular Disease. *Diabetes*. **64**, 3464-3474 (2015).
38 Milinkovic, V. *et al.* Identification of novel genetic alterations in samples of malignant glioma patients. *PLoS One*. **8**, e82108 (2013).
39 Waugh, M. G. Chromosomal Instability and Phosphoinositide Pathway Gene Signatures in Glioblastoma Multiforme. *Mol Neurobiol*. **53**, 621-630 (2016).
40 Sekulic, A. *et al.* Loss of inositol polyphosphate 5-phosphatase is an early event in development of cutaneous squamous cell carcinoma. *Cancer Prev Res (Phila)*. **3**, 1277-1283 (2010).
41 Gallagher, E. *et al.* Gain of imprinting of SLC22A18 sense and antisense transcripts in human breast cancer. *Genomics*. **88**, 12-17 (2006).
42 Travers, M. E. *et al.* Insights into the molecular mechanism for type 2 diabetes susceptibility at the KCNQ1 locus from temporal changes in imprinting status in human islets. *Diabetes*. **62**, 987-992 (2013).
43 Bajaj, V., Singhmar, P. & Kumar, A. Promoter characterization and regulation of expression of an imprinted gene SLC22A18AS. *Gene*. **424**, 40-47 (2008).
44 Sunamura, N. *et al.* Regulation of functional KCNQ1OT1 lncRNA by beta-catenin. *Sci Rep*. **6**, 20690 (2016).
45 Zhang, Z. *et al.* Long non-coding RNA chromogenic in situ hybridisation signal pattern correlation with breast tumour pathology. *J Clin Pathol*. **69**, 76-81 (2016).
46 Nilsson, E. *et al.* Altered DNA methylation and differential expression of genes influencing metabolism and inflammation in adipose tissue from subjects with type 2 diabetes. *Diabetes*. **63**, 2962-2976 (2014).
47 Vausort, M., Wagner, D. R. & Devaux, Y. Long noncoding RNAs in patients with acute myocardial infarction. *Circ Res*. **115**, 668-677 (2014).
48 Kornak, U. *et al.* Sensory neuropathy with bone destruction due to a mutation in the membrane-shaping atlastin GTPase 3. *Brain*. **137**, 683-692 (2014).
49 Guilmatre, A., Huguet, G., Delorme, R. & Bourgeron, T. The emerging role of SHANK genes in neuropsychiatric disorders. *Dev Neurobiol*. **74**, 113-122 (2014).
50 Devaney, J. M. *et al.* Genome-wide differentially methylated genes in prostate cancer tissues from African-American and Caucasian men. *Epigenetics*. **10**, 319-328 (2015).
51 Kolarova, J. *et al.* Array-based DNA methylation analysis in individuals with developmental delay/intellectual disability and normal molecular karyotype. *Eur J Med Genet*. **58**, 419-425 (2015).
52 Nordlund, J. *et al.* DNA methylation-based subtype prediction for pediatric acute lymphoblastic leukemia. *Clin Epigenetics*. **7**, 11 (2015).

53 Zhang, M. Y. *et al.* Germline ETV6 mutations in familial thrombocytopenia and hematologic malignancy. *Nat Genet*. **47**, 180-185 (2015).
54 Wang, X. & Chen, Z. MicroRNA-19a functions as an oncogenic microRNA in non-small cell lung cancer by targeting the suppressor of cytokine signaling 1 and mediating STAT3 activation. *Int J Mol Med*. **35**, 839-846 (2015).
55 Feng, Y. *et al.* miR-19a acts as an oncogenic microRNA and is up-regulated in bladder cancer. *J Exp Clin Cancer Res*. **33**, 67 (2014).
56 Giraldez, M. D. *et al.* Circulating microRNAs as biomarkers of colorectal cancer: results from a genome-wide profiling and validation study. *Clin Gastroenterol Hepatol*. **11**, 681-688 e683 (2013).
57 Yang, F. *et al.* MicroRNA-19a mediates gastric carcinoma cell proliferation through the activation of nuclear factor-kappaB. *Mol Med Rep*. **12**, 5780-5786 (2015).
58 Zhang, Y. *et al.* A systematic investigation based on microRNA-mediated gene regulatory network reveals that dysregulation of microRNA-19a/Cyclin D1 axis confers an oncogenic potential and a worse prognosis in human hepatocellular carcinoma. *RNA Biol*. **12**, 643-657 (2015).
59 Xu, X. M. *et al.* MicroRNA-19a and -19b regulate cervical carcinoma cell proliferation and invasion by targeting CUL5. *Cancer Lett*. **322**, 148-158 (2012).
60 Anfossi, S. *et al.* High serum miR-19a levels are associated with inflammatory breast cancer and are predictive of favorable clinical outcome in patients with metastatic HER2+ inflammatory breast cancer. *PLoS One*. **9**, e83113 (2014).
61 Lepore, I. *et al.* HDAC inhibitors repress BARD1 isoform expression in acute myeloid leukemia cells via activation of miR-19a and/or b. *PLoS One*. **8**, e83018 (2013).
62 Xu, X. L. *et al.* MicroRNA-17, microRNA-18a, and microRNA-19a are prognostic indicators in esophageal squamous cell carcinoma. *Ann Thorac Surg*. **97**, 1037-1045 (2014).
63 Zhong, J. *et al.* Circulating microRNA-19a as a potential novel biomarker for diagnosis of acute myocardial infarction. *Int J Mol Sci*. **15**, 20355-20364 (2014).
64 Nagaraj, S. H. & Reverter, A. A Boolean-based systems biology approach to predict novel genes associated with cancer: Application to colorectal cancer. *BMC Syst Biol*. **5**, 35 (2011).
65 Rafn, B. *et al.* ErbB2-driven breast cancer cell invasion depends on a complex signaling network activating myeloid zinc finger-1-dependent cathepsin B expression. *Mol Cell*. **45**, 764-776 (2012).
66 Kale, V. P. *et al.* A novel selective multikinase inhibitor of ROCK and MRCK effectively blocks cancer cell migration and invasion. *Cancer Lett*. **354**, 299-310 (2014).
67 Canto, E. *et al.* Validation of semaphorin 7A and ala-beta-his-dipeptidase as biomarkers associated with the conversion from clinically isolated syndrome to multiple sclerosis. *J Neuroinflammation*. **11**, 181 (2014).
68 Esnault, S. *et al.* Semaphorin 7A is expressed on airway eosinophils and upregulated by IL-5 family cytokines. *Clin Immunol*. **150**, 90-100 (2014).
69 De Minicis, S. *et al.* Semaphorin 7A contributes to TGF-beta-mediated liver fibrogenesis. *Am J Pathol*. **183**, 820-830 (2013).

70 Yoo, J. Y. *et al.* CRISPLD2 is a target of progesterone receptor and its expression is decreased in women with endometriosis. *PLoS One*. **9**, e100481 (2014).

71 Ono, R. *et al.* M-RIP, a novel target of JNK signaling and a requirement for human cancer cell invasion. *Int J Mol Med*. **22**, 199-203 (2008).

72 Gaillard, C. *et al.* Transcription and methylation analyses of preleukemic promyelocytes indicate a dual role for PML/RARA in leukemia initiation. *Haematologica*. **100**, 1064-1075 (2015).

73 Conway, K. *et al.* DNA methylation profiling in the Carolina Breast Cancer Study defines cancer subclasses differing in clinicopathologic characteristics and survival. *Breast Cancer Res*. **16**, 450 (2014).

74 Gauchotte, G. *et al.* Retinoid acid receptor expression is helpful to distinguish between adenoma and well-differentiated carcinoma in the thyroid. *Virchows Arch*. **462**, 619-632 (2013).

75 Vivekanandan, P. & Torbenson, M. Epigenetic instability is rare in fibrolamellar carcinomas but common in viral-associated hepatocellular carcinomas. *Mod Pathol*. **21**, 670-675 (2008).

76 Lund, A. H. miR-10 in development and cancer. *Cell Death Differ*. **17**, 209-214 (2010).

77 Hudson, J. *et al.* Overexpression of miR-10a and miR-375 and downregulation of YAP1 in medullary thyroid carcinoma. *Exp Mol Pathol*. **95**, 62-67 (2013).

78 Jia, H. *et al.* MicroRNA-10a is down-regulated by DNA methylation and functions as a tumor suppressor in gastric cancer cells. *PLoS One*. **9**, e88057 (2014).

79 Khan, S. *et al.* MicroRNA-10a is reduced in breast cancer and regulated in part through retinoic acid. *BMC Cancer*. **15**, 345 (2015).

80 Kohler, C. U. *et al.* Analyses in human urothelial cells identify methylation of miR-152, miR-200b and miR-10a genes as candidate bladder cancer biomarkers. *Biochem Biophys Res Commun*. **438**, 48-53 (2013).

81 Ohuchida, K. *et al.* MicroRNA-10a is overexpressed in human pancreatic cancer and involved in its invasiveness partially via suppression of the HOXA1 gene. *Ann Surg Oncol*. **19**, 2394-2402 (2012).

82 Sun, W., Ma, Y., Chen, P. & Wang, D. MicroRNA-10a silencing reverses cisplatin resistance in the A549/cisplatin human lung cancer cell line via the transforming growth factor-beta/Smad2/STAT3/STAT5 pathway. *Mol Med Rep*. **11**, 3854-3859 (2015).

83 Weiss, F. U. *et al.* Retinoic acid receptor antagonists inhibit miR-10a expression and block metastatic behavior of pancreatic cancer. *Gastroenterology*. **137**, 2136-2145 e2131-2137 (2009).

84 Xu, H. *et al.* Predictive Value of Serum miR-10b, miR-29c, and miR-205 as Promising Biomarkers in Esophageal Squamous Cell Carcinoma Screening. *Medicine (Baltimore)*. **94**, e1558 (2015).

85 Chen, B. S. & Li, C. W. Constructing an integrated genetic and epigenetic cellular network for whole cellular mechanism using high-throughput next-generation sequencing data. *BMC Syst Biol*. **10**, 18 (2016).

86 Al-Jamal, H. A. *et al.* Silencing of suppressor of cytokine signaling-3 due to methylation results in phosphorylation of STAT3 in imatinib resistant BCR-ABL positive chronic myeloid leukemia cells. *Asian Pac J Cancer Prev*. **15**, 4555-4561 (2014).

87 Calarco, A. *et al.* Role of SOCS3 evaluated by immunohistochemical analysis in a cohort of patients affected by prostate cancer: preliminary results. *Urologia*. **79 Suppl 19**, 4-8 (2012).
88 Chen, H. *et al.* Frequent SOCS3 and 3OST2 promoter methylation and their epigenetic regulation in endometrial carcinoma. *Am J Cancer Res*. **5**, 180-190 (2015).
89 Chen, X. *et al.* Analysis of DNA methylation and gene expression in radiation-resistant head and neck tumors. *Epigenetics*. **10**, 545-561 (2015).
90 Feng, Y. *et al.* SOCS3 promoter hypermethylation is a favorable prognosticator and a novel indicator for G-CIMP-positive GBM patients. *PLoS One*. **9**, e91829 (2014).
91 Kalmar, A. *et al.* DNA hypermethylation and decreased mRNA expression of MAL, PRIMA1, PTGDR and SFRP1 in colorectal adenoma and cancer. *BMC Cancer*. **15**, 736 (2015).
92 Kim, M. H. *et al.* Suppressor of cytokine signaling (SOCS) genes are silenced by DNA hypermethylation and histone deacetylation and regulate response to radiotherapy in cervical cancer cells. *PLoS One*. **10**, e0123133 (2015).
93 Niwa, Y. *et al.* Methylation silencing of SOCS-3 promotes cell growth and migration by enhancing JAK/STAT and FAK signalings in human hepatocellular carcinoma. *Oncogene*. **24**, 6406-6417 (2005).
94 Tokita, T. *et al.* Methylation status of the SOCS3 gene in human malignant melanomas. *Int J Oncol*. **30**, 689-694 (2007).
95 Wang, J. *et al.* SOCS3 methylation in synergy with Reg3A overexpression promotes cell growth in pancreatic cancer. *J Mol Med (Berl)*. **92**, 1257-1269 (2014).
96 Zhang, S., Wang, W., Wang, E. & Qiu, X. SOCS3 expression is inversely correlated with Pyk2 in non-small cell lung cancer and exogenous SOCS3 inhibits proliferation and invasion of A549 cells. *Pathology*. **44**, 434-440 (2012).
97 Chambers, J. C. *et al.* Epigenome-wide association of DNA methylation markers in peripheral blood from Indian Asians and Europeans with incident type 2 diabetes: a nested case-control study. *Lancet Diabetes Endocrinol*. **3**, 526-534 (2015).
98 Al Muftah, W. A. *et al.* Epigenetic associations of type 2 diabetes and BMI in an Arab population. *Clin Epigenetics*. **8**, 13 (2016).
99 Leger, A. J., Covic, L. & Kuliopulos, A. Protease-activated receptors in cardiovascular diseases. *Circulation*. **114**, 1070-1077 (2006).
100 Zhang, Y. *et al.* F2RL3 methylation, lung cancer incidence and mortality. *Int J Cancer*. **137**, 1739-1748 (2015).
101 Bonzheim, I. *et al.* CALR-mutated essential thrombocythemia evolving to chronic myeloid leukemia with coexistent CALR mutation and BCR-ABL translocation. *Blood*. **125**, 2309-2311 (2015).
102 Liu, X. *et al.* Anticancer effects of adenovirus-mediated calreticulin and melanoma-associated antigen 3 expression on non-small cell lung cancer cells. *Int Immunopharmacol*. **25**, 416-424 (2015).
103 Lu, Y. C., Weng, W. C. & Lee, H. Functional roles of calreticulin in cancer biology. *Biomed Res Int*. **2015**, 526524 (2015).
104 Shi, F. *et al.* Calreticulin promotes migration and invasion of esophageal cancer cells by upregulating neuropilin-1 expression via STAT5A. *Clin Cancer Res*. **20**, 6153-6162 (2014).

105 Dyer, M. J. & Oscier, D. G. The configuration of the immunoglobulin genes in B cell chronic lymphocytic leukemia. *Leukemia*. **16**, 973-984 (2002).

106 Lu, W. *et al.* MCPIP1 selectively destabilizes transcripts associated with an anti-apoptotic gene expression program in breast cancer cells that can elicit complete tumor regression. *Cancer Res* (2016).

107 Saamarthy, K. *et al.* Early diagnostic value of Bcl-3 localization in colorectal cancer. *BMC Cancer*. **15**, 341 (2015).

108 Dimitrakopoulos, F. I. *et al.* Variant of BCL3 gene is strongly associated with five-year survival of non-small-cell lung cancer patients. *Lung Cancer*. **89**, 311-319 (2015).

109 Ahlqvist, K., Saamarthy, K., Syed Khaja, A. S., Bjartell, A. & Massoumi, R. Expression of Id proteins is regulated by the Bcl-3 proto-oncogene in prostate cancer. *Oncogene*. **32**, 1601-1608 (2013).

110 Gao, Y., Qi, G. X., Guo, L. & Sun, Y. X. Bioinformatics analyses of differentially expressed genes associated with acute myocardial infarction. *Cardiovasc Ther* (2016).

111 Marchetti, G. *et al.* An integrated genomic-transcriptomic approach supports a role for the proto-oncogene BCL3 in atherosclerosis. *Thromb Haemost*. **113**, 655-663 (2015).

112 Meguro, K. *et al.* Role of Bcl-3 in the development of follicular helper T cells and in the pathogenesis of rheumatoid arthritis. *Arthritis Rheumatol*. **67**, 2651-2660 (2015).

113 Do, H. *et al.* A critical re-assessment of DNA repair gene promoter methylation in non-small cell lung carcinoma. *Sci Rep*. **4**, 4186 (2014).

114 Singh, K. P., Treas, J., Tyagi, T. & Gao, W. DNA demethylation by 5-aza-2-deoxycytidine treatment abrogates 17 beta-estradiol-induced cell growth and restores expression of DNA repair genes in human breast cancer cells. *Cancer Lett*. **316**, 62-69 (2012).

115 Wang, L. *et al.* Methylation markers for small cell lung cancer in peripheral blood leukocyte DNA. *J Thorac Oncol*. **5**, 778-785 (2010).

116 Ciaparrone, M. *et al.* Predictive Role of ERCC1 Expression in Head and Neck Squamous Cell Carcinoma Patients Treated with Surgery and Adjuvant Cisplatin-Based Chemoradiation. *Oncology*. **89**, 227-234 (2015).

117 Shen, J. *et al.* A three-gene signature as potential predictive biomarker for irinotecan sensitivity in gastric cancer. *J Transl Med*. **11**, 73 (2013).

118 Hassanein, M. *et al.* SLC1A5 mediates glutamine transport required for lung cancer cell growth and survival. *Clin Cancer Res*. **19**, 560-570 (2013).

119 Huang, F. *et al.* Upregulated SLC1A5 promotes cell growth and survival in colorectal cancer. *Int J Clin Exp Pathol*. **7**, 6006-6014 (2014).

120 Wang, Q. *et al.* Targeting glutamine transport to suppress melanoma cell growth. *Int J Cancer*. **135**, 1060-1071 (2014).

121 Wang, Q. *et al.* Targeting ASCT2-mediated glutamine uptake blocks prostate cancer growth and tumour development. *J Pathol*. **236**, 278-289 (2015).

122 Kaira, K. *et al.* Clinicopathological significance of ASC amino acid transporter-2 expression in pancreatic ductal carcinoma. *Histopathology*. **66**, 234-243 (2015).

123 Ren, P. *et al.* ATF4 and N-Myc coordinate glutamine metabolism in MYCN-amplified neuroblastoma cells through ASCT2 activation. *J Pathol*. **235**, 90-100 (2015).

124 Liu, Y. *et al.* High expression of Solute Carrier Family 1, member 5 (SLC1A5) is associated with poor prognosis in clear-cell renal cell carcinoma. *Sci Rep*. **5**, 16954 (2015).

125 van Geldermalsen, M. *et al.* ASCT2/SLC1A5 controls glutamine uptake and tumour growth in triple-negative basal-like breast cancer. *Oncogene* (2015).

126 Dayeh, T. *et al.* Genome-wide DNA methylation analysis of human pancreatic islets from type 2 diabetic and non-diabetic donors identifies candidate genes that influence insulin secretion. *PLoS Genet*. **10**, e1004160 (2014).

127 Honjo, Y. *et al.* Increased GADD34 in oligodendrocytes in Alzheimer's disease. *Neurosci Lett*. **602**, 50-55 (2015).

128 Morton, E., Macrae, I. M., McCabe, C., Brown, S. M. & White, F. Identification of the growth arrest and DNA damage protein GADD34 in the normal human heart and demonstration of alterations in expression following myocardial ischaemia. *Int J Cardiol*. **107**, 126-129 (2006).

129 Nishio, N. & Isobe, K. GADD34-deficient mice develop obesity, nonalcoholic fatty liver disease, hepatic carcinoma and insulin resistance. *Sci Rep*. **5**, 13519 (2015).

130 Tanaka, Y. *et al.* Effects of growth arrest and DNA damage-inducible protein 34 (GADD34) on inflammation-induced colon cancer in mice. *Br J Cancer*. **113**, 669-679 (2015).

131 Lee, D. I. *et al.* Phosphodiesterase 9A controls nitric-oxide-independent cGMP and hypertrophic heart disease. *Nature*. **519**, 472-476 (2015).

132 Karami-Tehrani, F., Moeinifard, M., Aghaei, M. & Atri, M. Evaluation of PDE5 and PDE9 expression in benign and malignant breast tumors. *Arch Med Res*. **43**, 470-475 (2012).

133 Sato, T. *et al.* DNA methylation profiles at precancerous stages associated with recurrence of lung adenocarcinoma. *PLoS One*. **8**, e59444 (2013).

134 Cheng, X. *et al.* MKL1 potentiates lung cancer cell migration and invasion by epigenetically activating MMP9 transcription. *Oncogene*. **34**, 5570-5581 (2015).

135 Luo, X. G. *et al.* Histone methyltransferase SMYD3 promotes MRTF-A-mediated transactivation of MYL9 and migration of MCF-7 breast cancer cells. *Cancer Lett*. **344**, 129-137 (2014).

136 Sisson, T. H. *et al.* Inhibition of myocardin-related transcription factor/serum response factor signaling decreases lung fibrosis and promotes mesenchymal cell apoptosis. *Am J Pathol*. **185**, 969-986 (2015).

137 Fan, Z. *et al.* MKL1 is an epigenetic modulator of TGF-beta induced fibrogenesis. *Biochim Biophys Acta*. **1849**, 1219-1228 (2015).

138 Horvath, S. DNA methylation age of human tissues and cell types. *Genome Biol*. **14**, R115 (2013).
